# BUILDing BLaST: promoting rural students’ biomedical research careers using a culturally responsive, one health approach

**DOI:** 10.1186/s12919-017-0092-7

**Published:** 2017-12-04

**Authors:** Barbara E. Taylor, Arleigh J. Reynolds, Kathy E. Etz, Nicole M. G. MacCalla, Paul A. Cotter, Tiffany L. DeRuyter, Karsten Hueffer

**Affiliations:** 1 University of Alaska Fairbanks Biomedical Learning and Student Training Program, Fairbanks, Alaska 99775 USA; 2 Department of Biology & Wildlife, University of Alaska Fairbanks, Fairbanks, Alaska 99775 USA; 3 Department of Veterinary Medicine, University of Alaska Fairbanks, Fairbanks, Alaska 99775 USA; 4 University of Alaska Fairbanks, Institute of Arctic Biology, Fairbanks, Alaska 99775 USA; 5 National Institute on Drug Abuse, Division of Epidemiology, Services and Prevention Research, National Institutes of Health, Bethesda, Maryland 20892 USA; 6 Department of Education, Graduate School of Education and Information Studies, University of California, California, Los Angeles 90095 USA; 7 EvaluLogic, Sitka, Alaska 99835 USA

## Abstract

**Background and purpose:**

Most postsecondary institutions in the state of Alaska (USA) have a broad mission to serve diverse students, many of whom come from schools in rural villages that are accessible only by plane, boat, or snowmobile. The major research university, the University of Alaska in Fairbanks (UAF), serves a population whereby 40% are from groups recognized as underrepresented in the biomedical workforce. The purpose of this article is to describe the Building Infrastructure Leading to Diversity (BUILD)-supported program in the state of Alaska that seeks to engage students from rural areas with a culturally relevant approach that is centered on the One Health paradigm, integrating human, animal, and environmental health.

**Program and key highlights:**

The Biomedical Learning and Student Training (BLaST) program distinguished by broad themes that address recruitment, retention, and success of students in biomedical programs, especially for students from rural backgrounds. Targeted rural outreach emphasizes that biomedical research includes research on the integration of human, animal, and environmental health. This One Health perspective gives personal relevance and connection to biomedical research. This outreach is expected to benefit student recruitment, as well as foster family and community support for pursuit of college degrees. BLaST promotes integration of research into undergraduate curricula through curriculum development, and by creating a new class of instructors, laboratory research and teaching technicians, who provide research mentorship, course instruction, and comprehensive advising. Finally, BLaST facilitates early and sustained undergraduate research experiences in collaborations with graduate students and faculty.

**Implications:**

BLaST’s approach is highly adapted to the Alaskan educational and physical environment, but components and concepts could be adapted to other rural areas as a means to engage students from rural backgrounds, who often have a closer relationship with the natural environment than urban students.

## Background and context

**Fig. 1:**
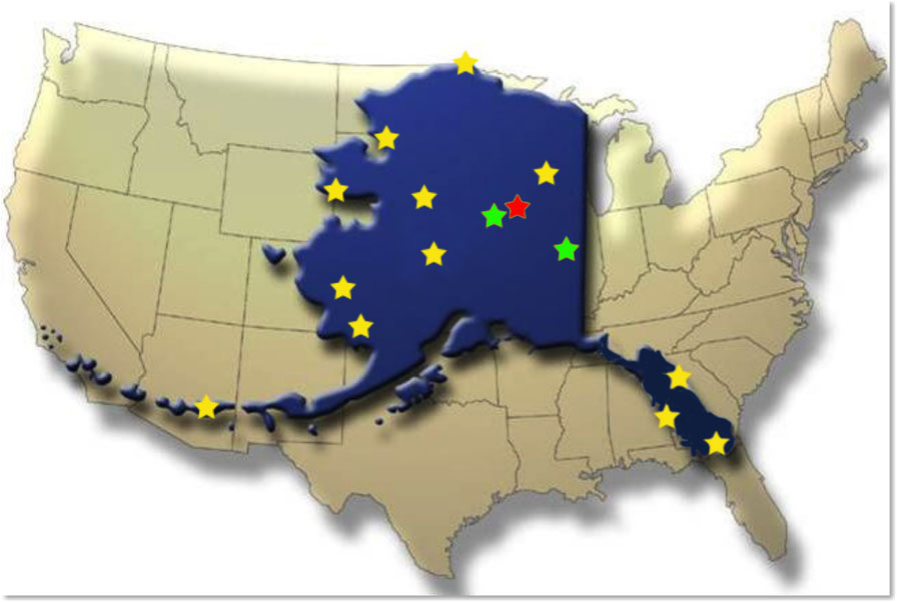
Alaska (blue overlay) in comparison to the contiguous United States. Location of the BLaST network are indicated by stars. The location of the Fairbanks campus is indicated in red. Partner locations with road access are indicated in green, while yellow stars indicate fly-in-only locations. The distance between the northern most location Utqiaġvik (formerly known as Barrow) and Ketchikan in the south is 2140 km

Education in the state of Alaska (USA) is defined by its remote location and size (Fig. 1). To prepare students, including those from rural areas, for biomedical research careers it is critical to understand Alaska’s education pipeline. Alaska residents make up 90% of the student population at University of Alaska Fairbanks (UAF). Of 503 K-12 schools in the state, about 25% have a total student enrollment of 50 or fewer. Half of Alaska’s schools are in small villages that are accessible only by plane, boat, or snowmobile; have three teachers for all grades combined; have 90–100% Alaska Native students; and serve 20% of the state’s students. Alaska Natives make up 23% of the state’s K-12 students, but account for 41% of drop-outs.

The purpose of this article is to describe the Building Infrastructure Leading to Diversity (BUILD)-supported program in the state of Alaska that seeks to engage students from rural areas with a culturally relevant approach that is centered on the One Health paradigm, integrating human, animal, and environmental health. UAF offers programs ranging from developmental education and lifelong learning to associate, bachelors, masters, and doctoral degrees. Despite a broad mission serving a diverse student population, UAF is a research university with a focus on science, technology, engineering, and mathematics (STEM) fields. Programs in STEM disciplines exist in biology, climate change, engineering, fisheries and ocean sciences, geology, geophysics, space physics, and wildlife biology. UAF offers 18 PhD programs (366 students) with a particular strength in the sciences, engineering, psychology, indigenous studies, and anthropology. There are over 50 masters programs (765 students), over 70 baccalaureate programs (3657 students), and over 25 associate degree programs (1396 students).

Undergraduate students at UAF differ in their demographics relative to students at comparable institutions. Based on fall 2015 data, the median age of UAF’s undergraduate students is 25. More UAF students (62%) attend part-time compared to the national mean (43%). Forty-one percent of UAF students complete their degree in 6 years, and only 40% who begin in a STEM program complete that degree. Mean numbers from 2007 to 2011 indicate that among 3514 students at the Fairbanks campus fitting the citizen or resident definition, 49% are low income or first generation college students and 5 % have a documented disability. Overall, 40% of UAF students are from groups recognized by the National Institutes of Health (NIH) as underrepresented in the biomedical workforce [1]. In planning meetings with faculty representatives from rural partner institutions prior to the development of the BLaST program, we identified several rural student needs that will be supported by the BLaST program, specifically acquisition of study skills, access to technology, and need for counseling assistance. Upon entry, 84% of students in UAF’s Student Support Services (SSS) program indicate they need help with study skills. Low-income students commonly report having limited access to technology and limited skills for its use.

Based on self-reported data, Alaska Natives compose about 20% of UAF’s student population, which matches the state population. Rural students in particular face many challenges. Most UAF students cannot drive home on weekends for support from family, friends, and role models. Rural communities often have few college graduates. Thus, many rural students will not have local role models with college experience and will be unable to receive guidance on academic success. Therefore, first generation college students are disproportionately fewer among students both attending and graduating from doctoral programs [2, 3]. While UAF and the University of Alaska Southeast (UAS) (a BLaST partner) enroll Alaska Native students in a similar proportion to the state population, many of these students enroll at rural campuses for certificate or professional endorsement programs. They also do not enroll in bachelors degree programs or at the Fairbanks and Juneau campuses (only 14% of students at those campuses are Alaska Native), where most research opportunities exist. Only 6% of doctoral recipients are Alaska Native, reflecting national trends [4] (Of 18,758 doctorate awardees in the US in 2009, only 120 were Alaska Native or American Indian (0.6%). The American Council on Education reports Native Americans between 1995 and 2005 comprised 0.3 to 0.4% of all doctorates granted [5].

Support services and awareness programs provide pathways for rural, underrepresented and, in particular, Alaska Native students to graduate in a timely fashion. The Rural Alaska Honors Institute (RAHI), Upward Bound (UB), Alaska Native Science and Engineering Program (ANSEP), Student Support Services (SSS), and American Indian Science and Engineering Society (AISES) programs strive to increase these student’s rates of success. These programs are successful despite UAF students from underrepresented groups commonly having high school grade point averages below 3.0, ACT scores less than 18, the need for developmental math and English courses prior to declaring a major, and being enrolled part-time. Even with these programs’ successes, additional support is necessary to ensure the success of rural students as these students still graduate at lower rates and continue into graduate programs less often than non-rural students.

## Integrating research and teaching

UAF has a historically poor record of integrating research and teaching activities, which is a common problem in research intensive institutions [6]. Faculty and administrators may not recognize the key role undergraduate students play in the campus’ research mission, and undergraduate research mentoring may not be recognized in tenure and promotion decisions. Additionally, split appointments of many UAF STEM faculty in research institutes and academic departments means the research infrastructure is not well integrated with the academic teaching infrastructure. The benefit of split appointments is that faculty research time is protected; however, the disadvantage is that research and teaching are poorly integrated, because they are the purview of separate administrative units that potentially have different goals. Thus, undergraduate research training commands little attention by at least some research administrators. Split appointments have emerged as a major barrier for development of research programs in biomedical science [7] especially for undergraduate research programs, which rely on the integration of research and teaching.

Faculty in rural institutions have an advantage in this regard. Most have a bipartite workload composed of teaching and service, so any research conducted must be integrated with teaching. Even faculty with assignments in research, service, and teaching have high teaching loads and are more pressured to pursue research by integrating it with teaching duties in rural contexts. BLaST coordinates programmatic changes to integrate teaching and research, thereby enhancing research training for all undergraduates, including rural students. Recently, UAF created organizational units that integrate teaching and research. In 2011 the office of Undergraduate Research and Scholarly Activity was created to promote undergraduate research experiences. In 2013 the Department of Veterinary Medicine was established as an academic biomedical department with a mission to fully integrate teaching and research. BLaST is housed in the Department of Veterinary Medicine and thereby provides significant visibility to the research efforts in this integrated department. Currently, the university has a vibrant culture of undergraduate research, where 42% of students participate in research. UAF is uniquely positioned to serve students from rural Alaska in a healthy, well-supported, and active research environment. Furthermore, UAF’s Vision 2017 and the 2012 Strategic Plan (see UAF’s planning documents [8, 9] emphasize undergraduate research because it is recognized as a high-impact practice that leads to improved student retention and completion, and is an excellent means to build skills in critical thinking and creative problem solving [10]. Moreover, recent studies suggest that undergraduate research significantly improves intentions to attend graduate school, and this is particularly impactful for underrepresented groups [11, 12].

Over the past decade, infrastructure building grants from the NIH (Centers of Biomedical Research Excellence (COBRE), the IDeA Network of Biomedical Research Excellence (INBRE), and the Specialized Neuroscience Research Program (SNRP)) have led to growth in biomedical research funding ($6 M in 2004 to $18 M in 2013 to $42 M in 2015). So the natural next step is to integrate student training with this infrastructure development. While BLaST harnesses existing infrastructure and works closely with existing student support programs, such as SSS, RAHI, and ANSEP, these efforts are distinct and innovative. Through close integration of research and teaching, we enhance research-training opportunities and better engage rural college campuses with efforts at the Fairbanks campus to improve opportunities for students through collaborative partnerships. We leverage existing faculty and infrastructure to improve visibility and enhance opportunities for competitive biomedical research with a focus on undergraduate training. Other programs in the past have not engaged rural college partner sites in the biomedical research enterprise. Therefore, BLaST provides a significant expansion of efforts to reach rural students throughout Alaska.

Recent advances in infrastructure, a unique student body, and the geographic location put UAF in a unique position to address the underrepresentation of students from rural backgrounds in biomedical programs.

## The one health paradigm for training

The rural subsistence lifestyle, which is focused on fishing, hunting, and gathering food from the environment, is a challenge for traditional western-based approaches of science and student engagement. By presenting biomedicine in the context of the One Health paradigm, we specifically engage and retain underrepresented students in this program that synergistically integrates research and teaching. In a subsistence culture, the environment and animals on the landscape provide food and subsistence activities that are culturally important to communities in rural Alaska. The One Health paradigm posits that the health of the environment, animals, and people are inextricably linked [13] and must involve a collaboration of professionals in the health, environmental and veterinary sciences working together to sustain the livelihood of all living beings. Examples of curricular topics that are relevant in Alaska, especially rural Alaska, include zoonotic diseases of wildlife such as rabies and tularemia, and contaminants in the food chain (e.g. mercury in marine mammals and fish resources) as well as the effects of environmental change on subsistence resources such as fish and wildlife. Students, especially those who attend partner institutions in rural areas, are well aware of environmental changes that affect whaling, economic activity, and cultural traditions, but may not understand the western based science behind the effects of climate change. Integrating these areas in a meaningful way that reflects and respects life experiences and cultural traditions, such as hunting and fishing for sustenance, brings biomedicine into the sphere of rural students’ experiences in remote Alaska. An approach where the environment, animals, and humans are interconnected is also better aligned with a more circular approach in indigenous knowledge than western science’s typically linear and reductionist approach [14]. Many of the students in these rural partner institutions are Alaska Natives.

## Research/pipeline partnerships

The BLaST network is a partnership between two separately accredited universities within the University of Alaska system (UAF and UAS) as well as Ilisagvik College, Alaska’s only accredited tribal college. Information on enrollment and student demographics is provided in Table 1. Over the years UAF developed rural campuses: the Community and Technical College in Fairbanks, Chukchi Campus in Kotzebue, Northwest Campus in Nome, Kuskokwim Campus in Bethel, Bristol Bay Campus in Dillingham, and the Interior Alaska Campus, which serves numerous communities in Interior Alaska through administrative and educational centers in Fairbanks, Fort Yukon, and Tok. UAS is a regional unit of the University of Alaska statewide system that also supports rural campuses located in Ketchikan and Sitka. The rural campuses of UAF and UAS are two-year colleges that serve as pipeline partners for the 4-year campuses in Juneau and Fairbanks. Ilisagvik is Alaska’s only tribal college located in Utqiaġvik (formerly known as Barrow), and also is an institution with a two-year focus. The location of the partners in the BLaST network are indicated by stars in Fig. 1.

**Table 1:** Enrollment information and student demographics for campuses of the BLaST network

	University of Alaska Fairbanks						University of Alaska Southeast			Ilisagvik College
Campus location	Bethel	Dillingham	Fairbanks	Interior	Kotzebue	Nome	Juneau	Sitka	Ketchikan	Barrow
Total headcount	354	889	9870	512	338	320	2910	1047	653	1666
Alaska Native/American Indian	62.7%	58.3%	10.4%	53.1%	48.2%	54.7%	14.1%	18.0%	14.9%	56.0%
Pacific Islander	0.6%	0.2%	0.7%	0	0.3%	0.6%	1.8%	1.5%	1.2%	–
Black	0	0.6%	2.3%	0.8%	2.7%	0.6%	0.9%	2.1%	1.4%	–
White	16.9%	29.7%	63.3%	33.0%	35.8%	25%	55.8%	55.8%	59.0%	–
Other	19.2%	10.9%	23.3%	12.9%	13.0%	18.8%	21.8%	22.6%	23.6%	–
Non-degree-seeking	39.9%	52.7%	12.1%	31.6%	58.1%	72.0%	20.8%	30.2%	24.6%	47.8
Associate degree	20.3%	33.0%	4.9%	42.6%	0.8%	2.2%	12.7%	9.6%	19.5%	52.2
Baccalaureate degree	1.8%	1.1%	66.2%	0.2%	0.6%	0.6%	45.3%	0	0	0

Data in this table were compiled from references [41–50]

## Program highlights: Preparing students for biomedical careers

Overall the BLaST program aims to:

- Emphasize active and experiential learning in a One Health paradigm. Activities include faculty training and support to develop and implement frequent classroom activities that require every student to engage in critical thinking and creative problem solving; and training and support for faculty to develop and implement research projects designed to engage undergraduate students in biomedical research.
- Fully integrate teaching and research, thereby immersing students in biomedical research throughout their undergraduate studies. This aim includes providing course-based research opportunities for lower division students with little or no background in college-level science, faculty-mentored projects as central components of upper division research courses, and summer research experiences.
- Embed students in a biomedical learning community that takes a holistic approach to student development by emphasizing cultural inclusiveness and comprehensive advising as well as training in critical-thinking and problem-solving skills. Activities to support this goal include establishing a biomedical learning community that integrates tiered group mentoring and ensures inclusion of students in face-to-face and online tutoring and research discussion activities that enhance biomedical knowledge, research skills, and ultimately students’ self-identification as biomedical professionals. Mentoring is a well-established means to promote student retention and success [15, 16].
- A unique aspect of our program is the integration of technical research staff in the mentoring of undergraduate students through the creation of Laboratory Research and Teaching Technician (LRTT) positions. In addition, we offer Graduate Mentoring Research Assistantships (GMRAs) that integrate undergraduate mentoring centrally in the graduate experience. These awards reflect our recognition that mentoring is part of holistic professional development for graduate students [17]. In addition, interaction with graduate students increases undergraduate students’ intentions to progress to graduate programs in STEM fields [18]. Our tiered group mentoring is defined as direct engagement of undergraduate students with a tiered group of mentors ranging from fellow undergraduate students to LRTTs, GMRAs, and faculty members.
- Develop and implement mentored research workshops and experiences at the rural partner institutions as well as the Fairbanks campus. These workshops and authentic, mentored experiences at different levels will serve as a first exposure to opportunities in biomedical research for many rural students within their communities and cultures.
- Develop and implement summer biomedical research experiences for rural students at the Fairbanks campus. This is accomplished with summer research modules: an assortment of research experiences at varying levels that accommodate a scaffolding of preparation, skills and knowledge that introduce students into the biomedical research enterprise.
- Establish the Active, Connected, and Experiential (ACE) learning initiative. ACE integrates high impact learning approaches at UAF. Students actively participate in introductory courses with research modules. They synthesize traditional course information, personally relevant life experience, and research experiences in newly developed One Health interdisciplinary courses, and they experience biomedical research through research-based courses.

## Evidence base for activity

The overarching goal of BLaST is to enhance the recruitment and retention of all Alaskan undergraduate students (including those from rural and Alaska Native backgrounds) into biomedicine-related degree programs and promote their successful advancement in biomedical research. To accomplish this goal, BLaST undertakes numerous interventions as indicated above. Many of these opportunities are offered to all students in biomedicine-related degree programs and many are required for BLaST scholars. BLaST scholars are selected annually through a rigorous application process that includes among other criteria diversity experiences. Scholars are funded through an award that includes tuition, fees, a monthly stipend, as well as funding for training and research costs. Such financial support is often required to facilitate and sustain enrollment for students, especially rural and Alaska Native students, at universities and colleges. Intensive mentoring and advising of the scholars by LRTTs, GMRAs and faculty within the tiered group mentoring approach helps develop this diverse talent pool represented by BLaST scholars.

## Undergraduate research and subsistence health

The BLaST program coined the term “Subsistence Health” to emphasize the importance of One Health in the context of a subsistence lifestyle. We take the well-developed One Health approach to research and public health and integrate it with student engagement and training in an innovative way that incorporates students’ own experiences and communities [19]. Such community involvement is integral to BLaST’s success because in rural Alaska, the bonds between people and place are strong, and western education is sometimes seen as a threat to the bonds of family and community and to the local culture [20–22].

Early engagement in research benefits both retention and success of undergraduate students [23–26]. BLaST offers undergraduate opportunities for early and sustained engagement in biomedical research to interested students through Undergraduate Research Experience awards, which support research costs and student salary. These awards are opportunities offered to undergraduate students who are not BLaST scholars and they reach a broader group of undergraduate students, including those at BLaST’s rural partner institutions. The engagement in research lasts from one semester to multiple years. Long-term research activities are encouraged to improve project ownership by the students.

BLaST facilitates early involvement in research through its development and promotion of courses in research readiness and integration of biomedical research modules into existing introductory courses. This curriculum development is primarily carried out by the LRTTs with oversight by the Principal Investigators. The LRTT positions are held by experienced teachers and researchers who hold masters or PhD degrees. This position is unique to BLaST and is a cornerstone of BLaST because the LRTTs are well positioned to bridge the divide between research and teaching. Early research involvement is also enabled by faculty buy-in to our program, particularly the willingness of faculty to accept research-naïve students into their projects, a willingness that is bolstered by the assurance that LRTTs will be on hand to assist with training and mentorship.

## Scaffolding and the use of LRTTs in mentoring

An extensive mentoring scaffold as represented by our tiered group mentoring approach is known to promote both retention and success of undergraduate students [27–30]. The LRTTs are especially important in the BLaST mentorship structure because they provide both mentorship and comprehensive advising to undergraduate students involved with BLaST. This advising includes intensive tutoring in class-related material and study skills, career advice, and direct research mentoring. These offerings are known to benefit American Indian and Alaska Native college students [31]. Each BLaST scholar is paired with a LRTT for comprehensive advising. LRTTs are available for comprehensive advising to all undergraduates participating in BLaST.

## Active learning and reflection

The active involvement in research is based on the experiential learning model [32], which postulates that students learn through active experimentation, which leads to a concrete experience. Subsequently, students reflect on their experiences and develop abstract concepts from reflective observations. Thus, active experimentation can be approached differently in subsequent rounds of learning. Many undergraduate research programs, including current efforts at UAF, focus on generating concrete experiences for students through participating in research projects that involve active experimentation on real scientific problems. The reflective observation and abstract conceptualization aspects of experiential learning are often under-emphasized. In our program we integrate these two key components into the undergraduate experience, thereby improving learning outcomes and academic success [32].

## Culturally responsive training

Honoring cultural elements shared by all Alaskans, especially those unique to rural Alaskans and Alaska Natives, and including those elements in our curricula creates an inclusive and comfortable community throughout our program. This is an evidenced-based approach that has worked well for indigenous communities throughout the US and Canada [33–37] and is working well for our program at UAF. BLaST’s community-building activities also include aspects of, and coordinate with, programs shown to successfully support rural and Alaska Native students and other diverse students. All these programs are known for providing tutoring, comprehensive advising, and student gathering centers that regularly hold cultural events. Thus, a culturally responsive learning community benefits recruitment and retention, especially for rural and Alaska Native students and students from other backgrounds that are underrepresented in biomedical careers.

## Institutional and faculty development

BLaST’s institutional and faculty development efforts enhance the research-training environment by enriching culture, institutional infrastructure, faculty skills, and mentoring capacity for biomedical education and research-training. This is facilitated by funding equipment purchases specifically designed to enable faculty to better engage undergraduate students in research training, as well as providing faculty with guidance on developing research projects that are amenable to undergraduate participation. Equipment purchases are proposed by faculty and selected by the BLaST program to enable student-focused research. Priority is given to equipment that will enable unique experiences in biomedical techniques to as many students as possible with a special focus on equipment for partner sites that will enable projects in rural Alaska. Furthermore, we logistically and financially support faculty as they develop instructional strategies, approaches, and skills to engage students and mentoring capabilities.

## Curricular innovations

BLaST has undertaken two categories of curriculum development activities. The first category is the ACE initiative, which includes active learning modules in credit-bearing introductory courses that connect research-training with the One Health conceptual coursework and provide training in professional skills and research techniques, and experiential learning through biomedical research participation. The second category consists of BLaST support for development of biomedical courses and course modules. These include massively open research experience courses focused on behavioral neuroscience that are suitable for distance delivery and are offered through the Fairbanks campus. Due to the dispersed nature of the Alaskan educational system, the distance delivery infrastructure is well developed even in the remote rural sites. BLaST, however, has also supported expansion of videoconference capabilities at the Fairbanks campus to increase interactions with rural partners.

Courses currently under development include five One Health courses that will be part of a One Health bachelors/masters program designed to accommodate students entering as freshmen or after having received an associate degree. The program will be offered at the Fairbanks campus and includes the following courses: a lower division course as an introduction to applied statistics, which will make statistics topically-relevant and tangible to the students interests; a middle division course will serve as an introduction to One Health, which will define the One Health paradigm using a broad approach with Alaska-relevant examples; and an upper division undergraduate course that is combined with a graduate level course on advanced topics in One Health. The latter course will provide an in-depth approach to the integrated nature of One Health issues with a focus on Alaska and an emphasis on how One Health issues arise and are resolved. Another upper division undergraduate course, taught in conjunction with a graduate level course as a One Health colloquium, will focus on critical evaluation of viewpoints of the many stakeholders with interests in a specific One Health problem, e.g. subsistence food availability, safety, and sustainability. Experts on several sides of the issues from multiple disciplines will present in a weekly seminar that will include discussions with, and questions from, students. At the conclusion of the course, students will, in small groups, develop and present in an open public forum a proposed process for management of the One Health issue studied during the semester. In addition to these courses at the Fairbanks campus, a microbiology course that integrates coursework and a research project on antibiotic resistance using a catheter sepsis model is currently offered at the Sitka campus.

In addition to course development, three new biomedical career pathways are being introduced into the curriculum. In the UAF College of Natural Science and Mathematics, a degree in Biology with a concentration in Biomedicine will be available for student enrollment starting spring semester 2017. This degree is intended to prepare students for biomedical graduate programs in research and health professions. Also in the college, a biomedical concentration in the biochemistry major will be offered beginning Fall 2017. This degree will incorporate research as an integral part of training during the first year and will incorporate a capstone project in the final year that can be expanded into a combined bachelors/masters degrees. This degree will prepare students to directly enter the biomedical workforce or proceed to graduate programs in biomedical research. The third biomedical degree option is being developed in conjunction with the College of Rural and Community Development. This will be a One Health concentration within the Rural Development bachelors degree program, it will allow students with an interest in this field to transfer in with an associates degree from our rural partner campuses and complete a bachelors degree within two to 3 years. This Rural Development bachelor degree with a One Health concentration is designed for students that may have difficulty meeting the pre-requisite criteria for standard biomedical curricula but have an interest in One Health as it pertains to rural Alaska. Students completing this degree will be prepared to advise rural communities on One Health issues and proceed to graduate school in One Health, community leadership, or the social sciences.

## Changes that likely lead to institutionalization of efforts and sustainability

Implementation of new courses and One Health programs will have an institutionalizing and sustainability effect by establishing the partners in the BLaST network as leaders in One Health education, especially in a rural context. This focus on One Health together with increased offerings in biomedical courses and research opportunities will boost UAF’s recruitment and lead to sustainability of the BLaST initiatives.

Comprehensive advising and tutoring will benefit student retention and completion, which will boost UAF’s performance as an institution of higher learning. This increased performance will lead to sustainability of these initiatives by creating institutional buy-in and long-term support, especially at rural partner institutions that are highly focused on student enrollment and success.

Faculty development as instructors, mentors, and biomedical researchers will foster “bottom-up” pressure or grassroots leadership to encourage administrators to work toward institutionalizing and sustaining BLaST initiatives. With increased faculty development that provides coaching for extramural research success, we expect such faculty to support sustaining efforts, especially when these initiatives lead to more faculty obtaining greater amounts of external funding. Some increases in applications for external funding and awards have already been seen as a result of these activities. In particular, a successful research grant was acquired by a faculty member from Ilisagvik College based on a BLaST-supported research program. This shows that research can be become sustainable with a student-focused approach and beneficial to faculty at small rural partner sites. Similarly, faculty-driven interest in LRTT positions will lead to sustained support for this approach of including technical staff in a tiered group mentoring approach to undergraduate education in research.

In addition, training graduate students and LRTTs in mentoring through the National Research Mentoring Network and similar programs, as well as including them as key members of a mentoring team, will increase the success of graduate students and technical staff in the academic enterprise, which is increasingly focused on undergraduate mentoring and greater diversity in STEM fields. Their BLaST experiences suggest that they will carry new knowledge, skills, and focus on undergraduate research with them throughout their careers in Alaska and elsewhere.

## Site-level evaluation design

The BLaST evaluation fits within larger Diversity Program Consortium’s efforts to identify mechanisms and strategies that are effective at increasing participation of underrepresented groups in biomedical research. Ideally, best practices from the BLaST experience can be exported to other programs for similar target populations or operating under similar challenges with rural populations. With a primarily formative and process evaluation focus, local evaluation for BLaST complements the Diversity Program Consortium’s overall impact evaluation efforts investigating student-, faculty-, and institutional-level effects. The local evaluation places less emphasis on comparison group strategies and more on longitudinal, pre-post, and retrospective pre-test approaches. The exact nature of the evaluation design varies based on stakeholder group and scope of program experience, which often varies based on geographic location. BLaST evaluation incorporates mixed method, qualitative, and quantitative approaches to capture process and formative as well as summative efforts at Fairbanks and rural partner campuses. Longitudinal trends in student-, faculty-, and institutional-level measures, and intervention-level assessments through retrospective pre-test and pre-post designs compose much of our quantitative evaluation efforts. Interviews, personal narratives, and reflective components connected to research projects compose much of our qualitative efforts.

Although we include all participating students, faculty/mentors, and institutions in evaluation efforts, data collection approaches (e.g. instrument design and deployment strategies) reflect cultural, geographic, demographic, and logistical variables across the BLaST landscape, especially as these variables pertain to students. Students attending the Fairbanks and Juneau campuses experience a more conventional collegiate academic and social environment than do those attending the other, smaller campuses. These differences are reflected in the strategies that we propose to capture student experience and formative input across campus types. Evaluating student experience at smaller, rural campuses deviates from those at the two larger campuses in the ratio of qualitative/quantitative data collected, and data collection strategies and instruments. Our previous experience suggests that approaching evaluation of research participants at smaller rural sites, and effectively capturing their experience through a programmatic-level lens, will neither sufficiently reflect their experience nor provide much-needed field input to inform program improvement. Additionally, modifications to semi-structured interviews for faculty are needed to help capture relevant topics including: promoting administrative support, utilizing the various BLaST resources, local recruitment strategies, and community engagement efforts at small rural campuses. Rural data are examined both in aggregate with, and contextually-separate from, program-wide data.

Differences between the two larger campuses and the smaller rural campuses necessitate modest modifications to the evaluation plan. To accomplish this, we adopt principles of utilization focused evaluation [38] and participatory action research [39]. Including and empowering high interest/low power stakeholders [38] both incentivizes participation in the evaluation development and provides a personally relevant mechanism to inform program direction [39]. In practice, both faculty/mentors and undergraduate researchers become co-researchers in the evaluation as they develop an appropriate format for the student narrative (including: journaling, blogging, social media projects, digital storybooks, audio narrative, etc.), and use data collection strategies that fit the everyday experience of the participants that capture experience, impact, and community relevance [40]. We anticipate that rural student engagement in evaluation development may highlight demographic-specific issues important for BLaST and other biomedical research-training initiatives with a rural focus.

Our local evaluation plan is built within the DPC’ Hallmarks of Success framework approved by all BUILD sites and addresses student, faculty/mentor, and institutional activities, outputs (e.g. research training, opportunities, and participation for students and faculty), and short-term outcomes (e.g. research knowledge and skills, and knowledge of biomedical career path progression for students; development of mentor and grant writing skills for faculty), medium - term outcomes (e.g. science identity, persistence, and research productivity for students; quality mentoring, collaborative curriculum development, and research productivity for faculty), and long-term outcomes (e.g. research productivity for students and faculty). Assessment of these outcomes is accomplished through targeted surveys, self-assessments, content knowledge assessments, interviews, and program benchmarking (see McCreath et al. in this issue for Consortium-wide Hallmarks of Success). Institutional-level goals assessed by the local evaluation include increasing research capacity at institutions, increasing diversity in biomedical disciplines, and strengthening pipelines to biomedical careers. Further, we aim to compare student-level pre-post responses with retrospective pre-test responses on self-assessments to address validity and reliability concerns in intervention-level evaluation activities.

Initial quantitative and qualitative data from students and faculty indicate high levels of engagement and satisfaction with mentored research experiences in BLaST. Undergraduate researchers report significantly increased interest, comfort, and competency in laboratory research, and improved understanding of science and of laboratory research methods (*p* < 0.01 in all cases; Wilcoxon Paired Sample Tests). Further, these improvements were observed each semester, as students continue to perceive learning in undergraduate research experiences through successive semesters of participation. Trends suggest undergraduate researchers’ interest in One Health topics increases following their research experiences; rural students are especially interested in connections between environment, animal, and human health. Process evaluation activities reveal that the broad reach of BLaST (urban/rural students across expansive geographic and cultural landscapes) requires well-defined organizational reporting structures and formalization of intra-campus partnerships to enhance program efficacy. Early identification of administrative limitations has enhanced program effectiveness and promoted leveraging of university resources to support wide-ranging BLaST initiatives.

## Conclusion: Unique features, potential contributions, and challenges

The unique features of BLaST include the use of the One Health concept in a locally adapted form as Subsistence Health in Alaska. The BLaST program proposes to engage a population underrepresented in the biomedical workforce by using a One Health focus to make biomedicine personally and culturally relevant, by equating biomedicine with the relationship between animal, human, and environmental health, which is a concept that resonates with Alaska Native and rural Alaskan culture. Another unique aspect of our program is a tiered group mentoring approach that specifically includes technical staff. By incorporating teaching and mentoring in the responsibilities of the LRTTs, we formalize the important role technical staff play in daily mentoring of undergraduate students in research. This formal recognition will increase the visibility of undergraduate research mentoring as an integral part of many technical employees beyond the LRTTs and will uniquely advance research mentoring in the BLaST network.

Potential contributions of BLaST to the broader academies of higher education include strategies for engaging, recruiting, and retaining rural students, especially those from backgrounds underrepresented in STEM fields, widespread adoption of the LRTT position, and culturally responsive mentoring for Alaska Native and American Indian students.

BLaST faces three important challenges. First the small numbers of students at rural campuses within the BLaST network will hamper quantitative and statistical analyses of BLaST’s outcomes and successes. Second, travel within Alaska and out of state is extremely costly and logistically challenging, especially for the rural college partner sites. Third, research is typically not a part of the academic culture at rural campuses, with most faculty having appointments dedicated to teaching and service. Nonetheless, we believe that other campuses serving underrepresented groups in rural areas may learn from, and adopt, the BLaST approach to enhancing research-training for biomedical careers.

## Abbreviations

ACE: Active, connected, and experiential
AISES: American indian science and engineering society
ANSEP: Alaska native science and engineering program
BLaST: Biomedical learning and student training
BUILD: Building infrastructure leading to diversity
CoBRE: Centers of biomedical research excellence
GMRA: graduate mentoring research assistantship
INBRE: IDeA network for biomedical research excellence
LRTT: laboratory research and teaching technician
NIH: National Institutes of Health
RAHI: Rural alaska honors institute
SNRP: Specialized neuroscience research program
SSS: Student support services
STEM: science, technology, engineering, and mathematics
UAF: University of Alaska Fairbanks
UAS: University of Alaska Southeast
UB: Upward bound
